# Prognostic value of CCR2 as an immune indicator in lung adenocarcinoma: A study based on tumor‐infiltrating immune cell analysis

**DOI:** 10.1002/cam4.3931

**Published:** 2021-05-04

**Authors:** Yi Wan, Xin Wang, Ting Liu, Tianyu Fan, Zugui Zhang, Bin Wang, Bei Zhang, Zibin Tian, Tao Mao, Zheng Gong, Li Zhang

**Affiliations:** ^1^ Department of Immunology School of Basic Medicine Qingdao University Qingdao Shandong Province China; ^2^ School of Stomatology Qingdao University Qingdao Shandong Province China; ^3^ Department of Gastroenterology The Affiliated Hospital of Qingdao University Qingdao Shandong Province China; ^4^ Value Institute Christiana Care Health System Newark DE USA; ^5^ Department of Specialty Medicine School of Basic Medicine Qingdao University Qingdao Shandong Province China; ^6^ Sino‐Cellbiomed Institutes of Medical Cell & Pharmaceutical Proteins Qingdao University Qingdao Shandong Province China

**Keywords:** CCR2, lung adenocarcinoma, prognosis, TCGA, tumor‐infiltrating immune cells

## Abstract

**Background:**

Prognostic indicators in lung adenocarcinoma (LUAD) have been seeking under database analysis, and remarkable advance is on the way.

**Methods:**

This study calculated the scores of stromal and immune components of the tumor microenvironment (TME) in 551 LUAD samples using the ESTIMATE algorithm on The Cancer Genome Atlas (TCGA) database. R package ''limma'' was used to selected differentially expressed genes (DEG). We have analyzed the DEGs by means of Gene Ontology (GO) analysis and Kyoto Encyclopedia of Genes and Genomes (KEGG) enrichments. The protein‐protein network, univariate Cox analysis, and Lasso regression analysis were performed to selected survival‐related genes. Gene Set Enrichment Analysis (GSEA) represented the enriched pathway of CC chemokine receptor 2 (CCR2). The ratios of immune cells in the TME of each LUAD sample were obtained using the R package "limma" and CIBERSORT algorithm in R 4.0.2.

**Results:**

The ImmuneScore was positively correlated with prognosis regarding survival rate, T classification of TNM stages, and clinicopathological staging characteristics. GO and KEGG enrichments showed DEGs were associated with immune‐related activities. Three genes of LUAD were selected from the PPI network and Cox proportional hazards regression analysis. CCR2 was the most survival correlated gene by Lasso regression analysis. GSEA results showed that C2 kegg gene sets in the CCR2 high‐expression group were mainly enriched in the B cell or T cell receptor signaling pathway and natural killer cell‐mediated cytotoxicity. Correlation of CCR2 expression with prognosis was conducted, implicating a positive correlation with the prognosis of survival rate and M classification, negative correlation with the prognosis of T and N classifications. The correlation between CCR2 and tumor‐infiltrating immune cells (TICs) was analyzed, and 14 kinds of TICs were found closely correlated with CCR2 expression through difference analysis.

**Conclusion:**

Therefore, CCR2 has prognostic value as an immune indicator in LUAD.

## Graphical abstract

The violin diagram showed nine kinds of TICs were positively correlated with CCR2 expression, including memory B cell , CD8+ T cells, gamma delta T cells, monocytes, resting and actived T cells memory, macrophage M1, resting dendritic cells, and resting mast cells; five kinds of TICs were negatively correlated with CCR2 expression, including follicular helper T cells, activated NK cells, macrophage M0, activated dendritic cells, and activated mast cells. These results further supported that the levels of CCR2 affected the immune activity of TME.

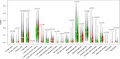

## 1 INTRODUCTION

Lung cancer, an extremely heterogeneous disease, causes almost a quarter of cancer‐related deaths.^1^ Lung adenocarcinoma (LUAD), accounting for about 40% of lung cancer, as a member of non‐small cell lung cancer (NSCLC), is the most aggressive histological type, and the incidence rises rapidly.^2^ Traditional treatment strategies for LUAD mainly focused on the strong dependence of tumor cells. In addition to cancer cells, there are immune cells, fibroblasts, endothelial cells, stromal cells, cytokines, chemokines, and receptors in TME, significantly influencing therapeutic effects.^3^ Tumor‐infiltrating immune cells (TICs) are promising indicators of immunotherapy in TME.^4^ The tumor‐infiltrating lymphocytes (TILs) were significantly correlated with the 5‐year survival of NSCLC, and low lymphocyte abundance in cancer was identified as a poor prognostic indicator in early‐stage NSCLC.^5^, ^6^ Considering the prognostic significance of TILs and other immune cells, a better understanding of the recruitment mechanism into the tumor is critical for good prognosis.

In this study, the ImmuneScore and StromalScore of LUAD data from the TCGA database were obtained through the ESTIMATE algorithm,^7^ an innovative algorithm to estimate stromal cells and immune cells in malignant tumor tissues by using tissue transcriptional profiling data. The algorithm calculates stromal and immune cells’ infiltration degree according to the specific gene expression characteristics. DEGs between tumor and normal tissues were selected, and the potential correlation between DEGs and immune‐related activities was studied by GO and KEGG enrichment analyses. Three genes were derived through the PPI network and Cox proportional hazards regression analysis from DEGs, among which CCR2 was the best choice as a predictive factor for prognosis. CCR2 is the chemokine mediating its biological effects through the G protein signaling pathway,^8^ expressed in various cells, consisting of monocytes, dendritic cells (DCs), endothelial cells, and cancer cells.^9^, ^10^, ^11^ The CCR2 and its ligand CCL2 signaling axis are involved in cancer pathogenesis by recruiting immune cells to tumor sites, thereby mediating various immune responses.^8^, ^12^, ^13^ To identified CCR2 as an excellent prognostic indicator, the correlation of CCR2 with immune response, prognosis, and TICs were also analyzed in this research.

## 2 MATERIALS AND METHODS

### 2.1 Data preparation

The gene‐expressed data (551 cases: 497 tumor cases and 54 normal cases, workflow type: HTseq‐FPKM, disease type: LUAD) and related clinical information were downloaded from the TCGA database (https://portal.gdc.cancer.gov/). After deleting the incomplete clinical information cases, there were 486 cases left (Table 1).

**TABLE 1:** Clinicopathological characteristics statistics in LUAD patients from TCGA

Clinical characteristics	Total (486)	%
Age at diagnosis (year)		
Young age (<=60)	155	31.9
Old age (>60)	312	64.2
Unknown	19	3.9
Gender		
Male	222	45.7
Female	264	54.3
Stage		
Ⅰ	262	53.9
Ⅱ	112	23.1
Ⅲ	79	16.3
Ⅳ	25	5.1
Unknown	8	1.6
T classification		
T1	163	33.6
T2	260	53.5
T3	41	8.4
T4	19	3.9
Unknown	3	0.6
N classification		
N0	312	64.2
N1	90	18.5
N2	70	14.4
N3	2	0.4
Unknown	12	2.5
M classification		
M0	333	68.5
M1	24	4.9
Unknown	129	26.6

### 2.2 Generation of Immune/Stromal/ESTIMATE Score and acquisition of DEGs

We utilized the R package "estimate"^7^ to calculate the proportion of immune and stromal elements in TME of tumor cases. This study expressed them in three scoring forms: ImmuneScore, StromalScore, and ESTIMATEScore. The scores were proportional to the proportion of the corresponding components in TME. The ESTIMATEScore was a comprehensive score of immune and stromal elements, meaning the combined ratio of the two elements in TME. In the ImmuneScore and StromalScore, a higher score indicated more immune or stromal elements in TME. Besides, 486 LUAD cases were divided into high and low scores, according to the ImmuneScore or StromalScore's median score. DEGs were obtained through difference analysis of gene expression using the R package "limma."

### 2.3 GO and KEGG enrichment analyses of DEGs

The 374 DEGs were used to perform GO and KEGG enrichment analyses by R 4.0.2 and the R packages "enrich plot," "Cluster Profiler," "ggplot2," and "org. Hs.eg.db." Only terms with both *p*‐ and *q*‐value <0.05 were considered significantly enriched.

### 2.4 PPI network construction and statistical analysis of DEGs

We built the PPI network in the STRING website (https://string‐db.org/), and then visualized the network using the Cytoscape version 3.7.2 software. Cox proportional hazards regression and Kaplan–Meier survival analyses were carried out on the 374 DEGs using the R package "survival." HR >1 indicated a high‐risk gene, and HR <1 indicated a low‐risk gene. The *p* < 0.01 was considered statistically significant.

### 2.5 Lasso regression analysis

The input files (patient survival time, survival state, and gene expression of each sample) were prepared, and the Lasso regression method was adopted to construct the multigene model using the R package "glmnet" and "survival".

### 2.6 GSEA enrichment analysis

The gene expression matrix file and cls file of group description were used as input files. The C7 gene set v7.1 and C2 kegg gene set v7.1 were selected as two main gene sets. All gene sets permuted 1000 times for each analysis and enriched in the pathway of NOM *p* < 0.05, |NES| > 1, FDR *q* < 0.05 were considered statistically significant.

### 2.7 Data analysis

All statistical tests were carried out by R 4.0.2. The relationship between the survival rate of LUAD cases and Immune/Stromal/ESTIMATE Score was calculated by Kaplan–Meier survival analysis. Wilcoxon rank test or Kruskal–Wallis rank‐sum test was utilized to analyze the correlation between clinicopathological characteristics (stage, muscular infiltration, lymph node status, distant metastasis, gender, and age) and Immune/Stromal/ESTIMATE Score. Wilcoxon rank‐sum or Kruskal–Wallis rank‐sum test was used to compare the effect of CCR2 expression on survival and other clinicopathological characteristics.

## 3 RESULTS

### 3.1 Correlation between Immune/Stromal/ESTIMATE Score and survival

**FIGURE 1:**
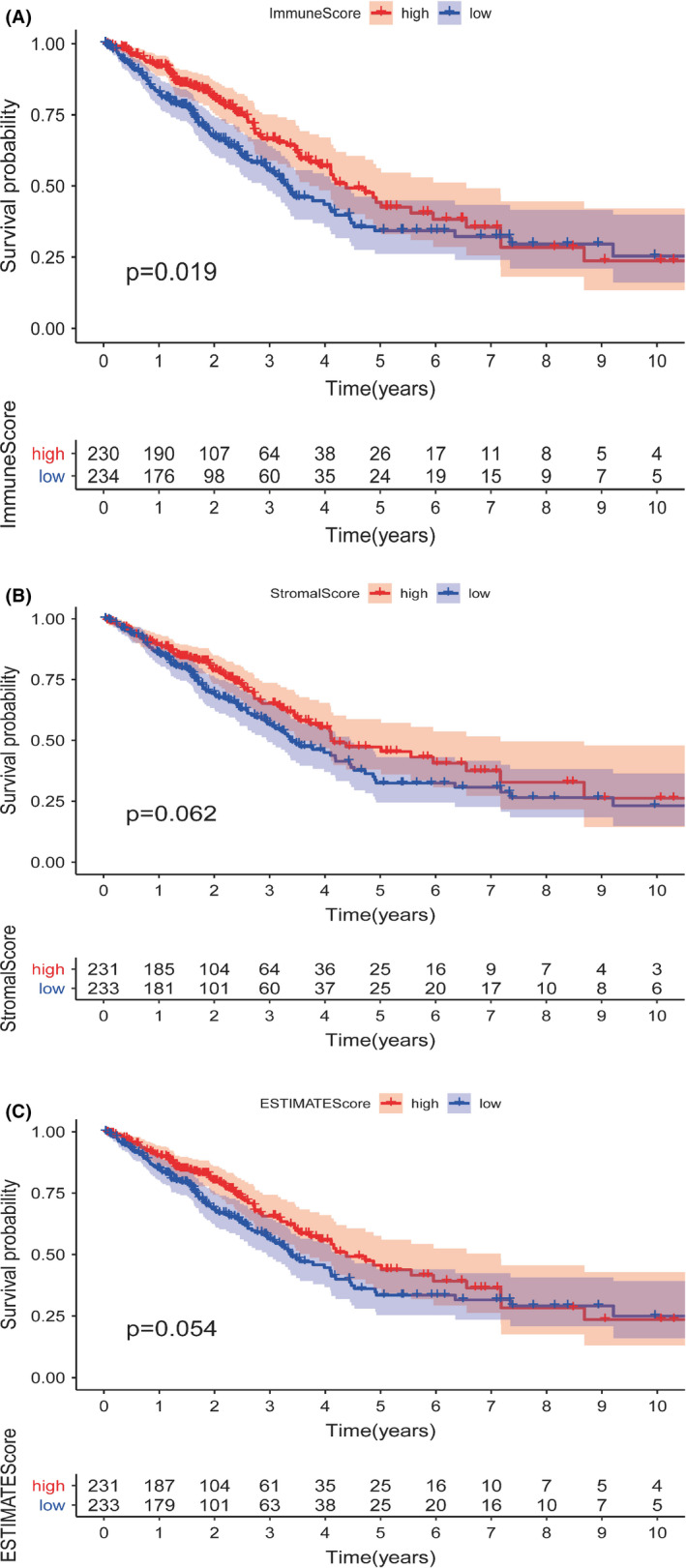
Correlation between Immune/Stromal/ESTIMATE Score and survival rate. (A) Kaplan–Meier survival analysis for ImmuneScore with *p* = 0.019 by Log‐Rank test. (B) Kaplan–Meier survival analysis for StromalScore with *p* = 0.062 by Log‐Rank test. (C) Kaplan–Meier survival analysis for ESTIMATScore with *p* = 0.054 by Log‐Rank test

486 LUAD cases were divided into high and low scores, according to the ImmuneScore or StromalScore's median score. Kaplan–Meier survival analysis was performed for immune and stromal components. The results implicated that the ImmuneScore was positively correlated with the survival rate (Figure 1A), while StromalScore and ESTIMATEScore were not correlated with the survival rate (Figure 1B and C).

### 3.2 ImmuneScore was correlated with TNM (Tumor, Lymph node, Metastasis) stages and clinicopathological characteristics

**FIGURE 2:**
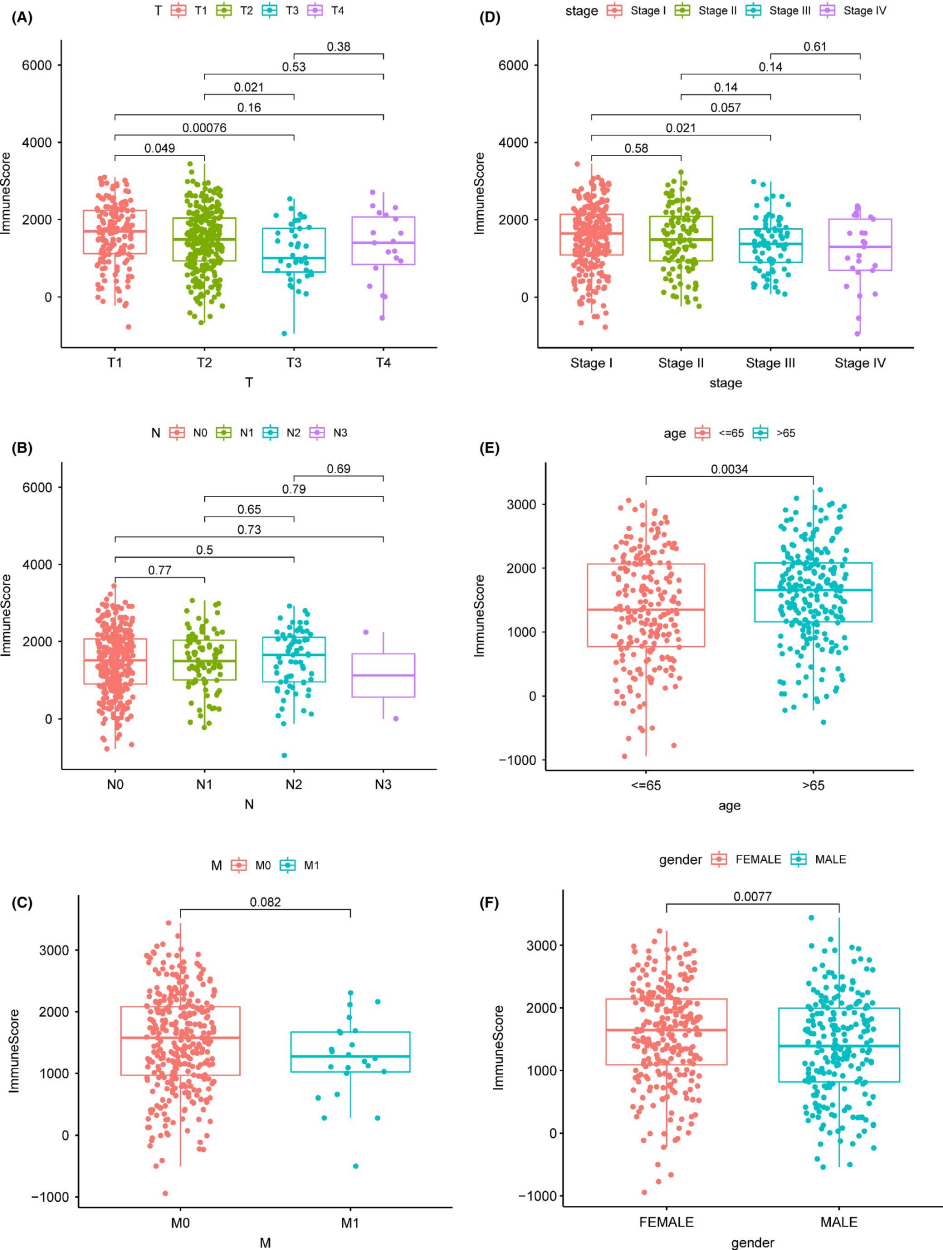
Correlation of ImmuneScore with TNM stages and clinicopathological characteristics. (A) Kruskal–Wallis rank‐sum test revealed ImmuneScore in different T classification and their correlation. (B) Kruskal–Wallis rank‐sum test revealed ImmuneScore in different N classification and their correlation. (C) Wilcoxon rank‐sum test revealed ImmuneScore in different M classification and their correlation. (D) Kruskal–Wallis rank‐sum test revealed ImmuneScore in different clinical stages and their correlation. (E) Wilcoxon rank‐sum test revealed ImmuneScore in different genders and their correlation. (F) Wilcoxon rank‐sum test revealed ImmuneScore in different ages and their correlation

The ImmuneScore was positively correlated with the T classification of TNM stages from T1 to T2, T3, and T4 (Figure 2A), while there was no significant correlation with N and M classifications (Figure 2B and C). The ImmuneScore was positively correlated with clinicopathological staging characteristics from Stage Ⅰ to Stage Ⅲ and Ⅳ (Figure 2D). Additionally, the ImmuneScore also correlated with age and gender (Figure 2E and F).

### 3.3 DEGs shared by ImmunoScore and StromalScore were mainly enriched in immune‐related activities

**FIGURE 3:**
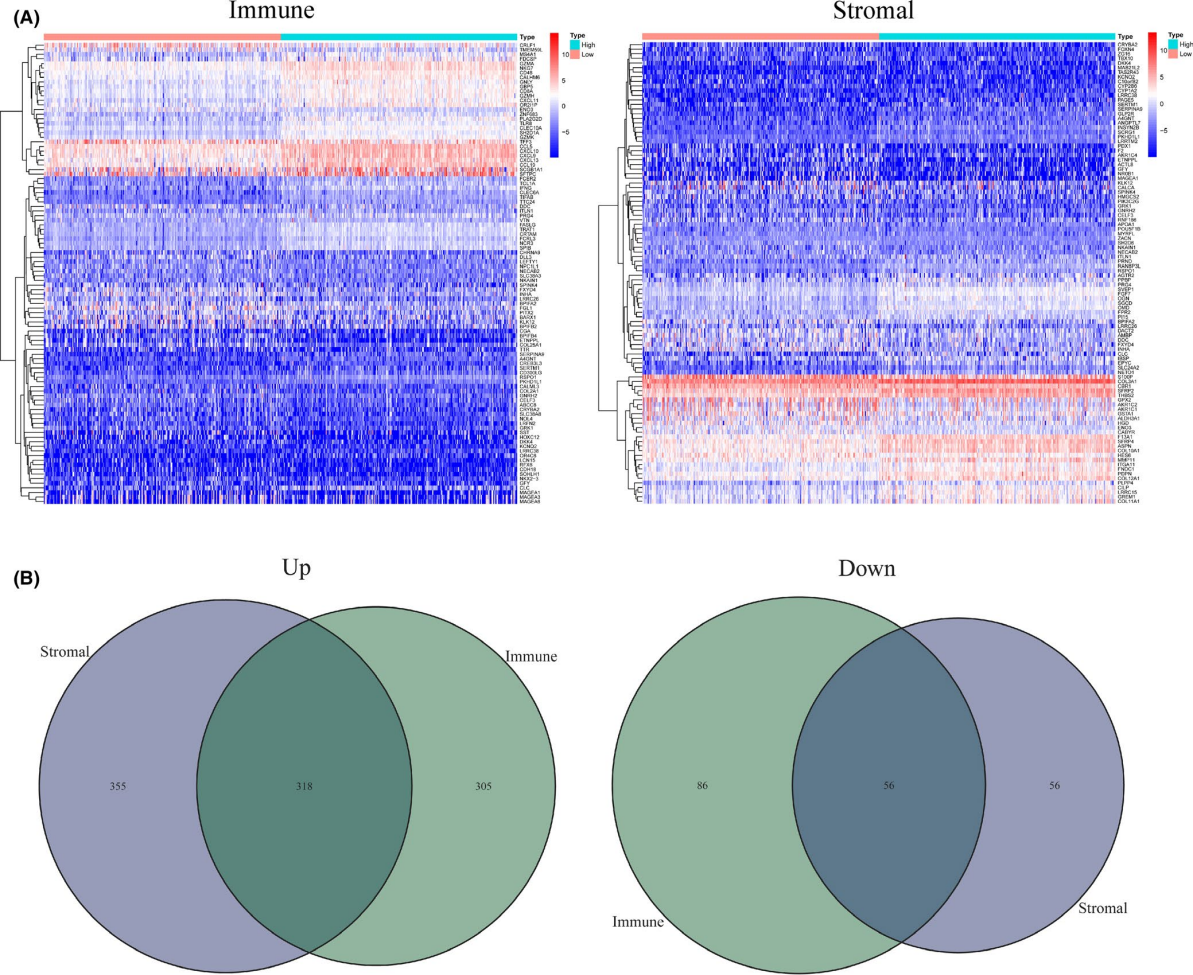
Heatmaps and Venn plots of DEGs. (A) The top 50 upregulated DEGs and the top 50 downregulated DEGs were selected according to absolute values logFC, and shown in each heat map. The DEGs were determined by the Wilcoxon rank‐sum test with *q* < 0.05 and fold‐change (FC) >1 after *log*2 transformation as the significance threshold. (B) Venn plots showed common 318 upregulated and 56 downregulated DEGs shared by ImmuneScore and StromalScore

**FIGURE 4:**
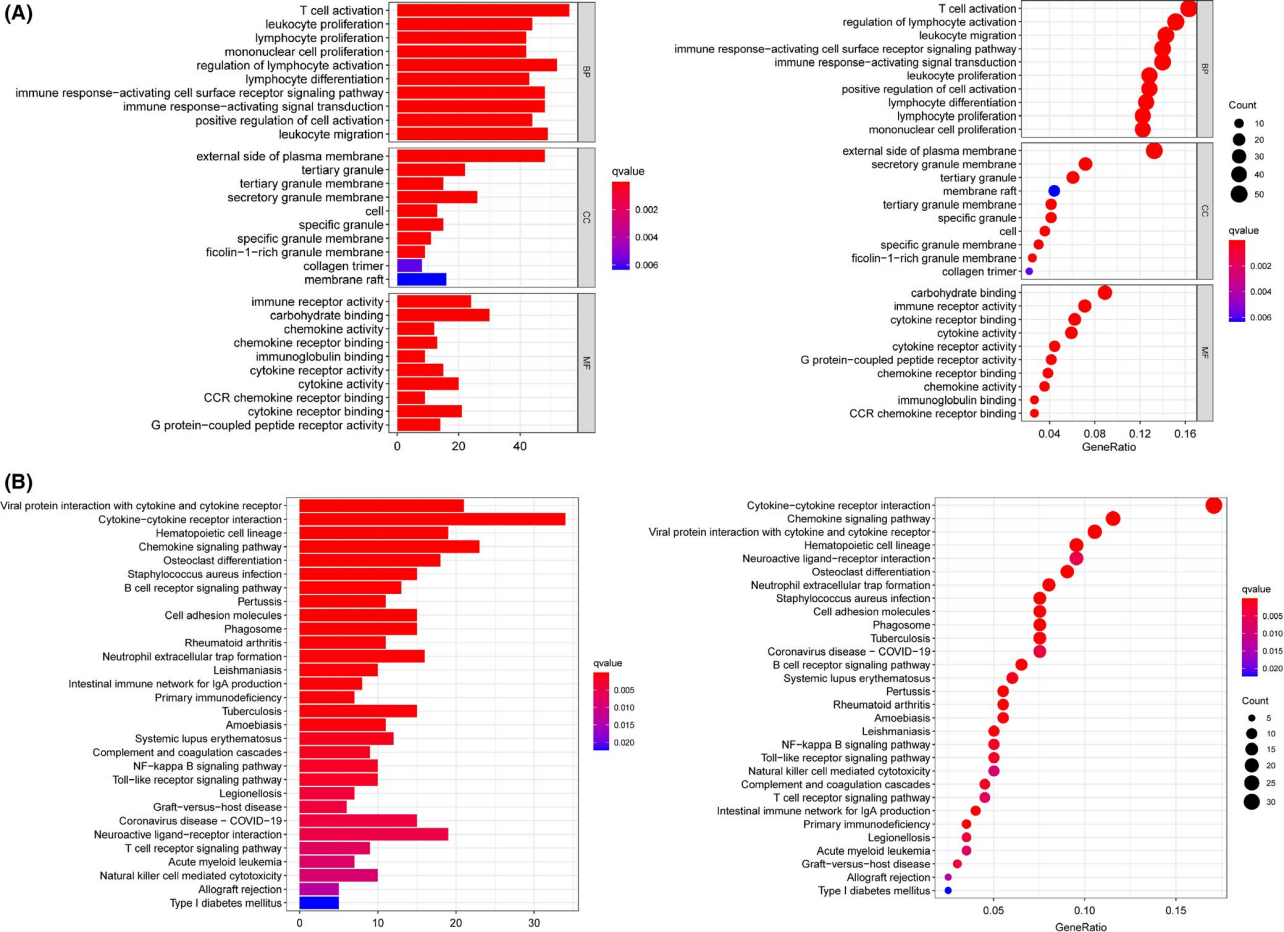
GO and KEGG enrichment. (A) Gene enrichment in three different GO functions and (B) KEGG pathways were respectively ranked by *q*‐value and gene enrichment count

The differences between high and low score groups in DEGs were compared, and results indicated a significant difference in the gene spectrum. We gained 765 genes from ImmuneScore, including 623 upregulated genes and 142 downregulated genes. Similarly, a total of 785 genes were obtained from StromalScore, including 673 upregulated genes and 112 downregulated genes (Figure 3A). Effective DEGs were overlap genes in both stromal and immune groups, and a total of 374 common differential genes were obtained by the R package "VennDiagram" in R 4.0.2, including 318 up and 56 downregulated genes (Figure 3B). Besides, the 374 DEGs were enriched in three different GO categories, such as the activating T cells, the immune receptor activity, and the outside of the plasma membrane, which were the most significant term in the biological process (BP), the molecular function (MF), and the cell component (CC) category, respectively (Figure 4A and Figure S1A). In the 374 DEGs, the top 3 KEGG terms were cytokine‐cytokine receptor interaction, chemokine signaling pathway, and viral protein interaction with cytokine by KEGG enrichment analysis (Figure 4B and Figure S1B). Both results of GO and KEGG enrichments predicted the potential correlation between DEGs and immune‐related activities.

### 3.4 Three significant prognostic genes in LUAD derived from PPI network and Cox proportional hazards regression analysis

**FIGURE 5:**
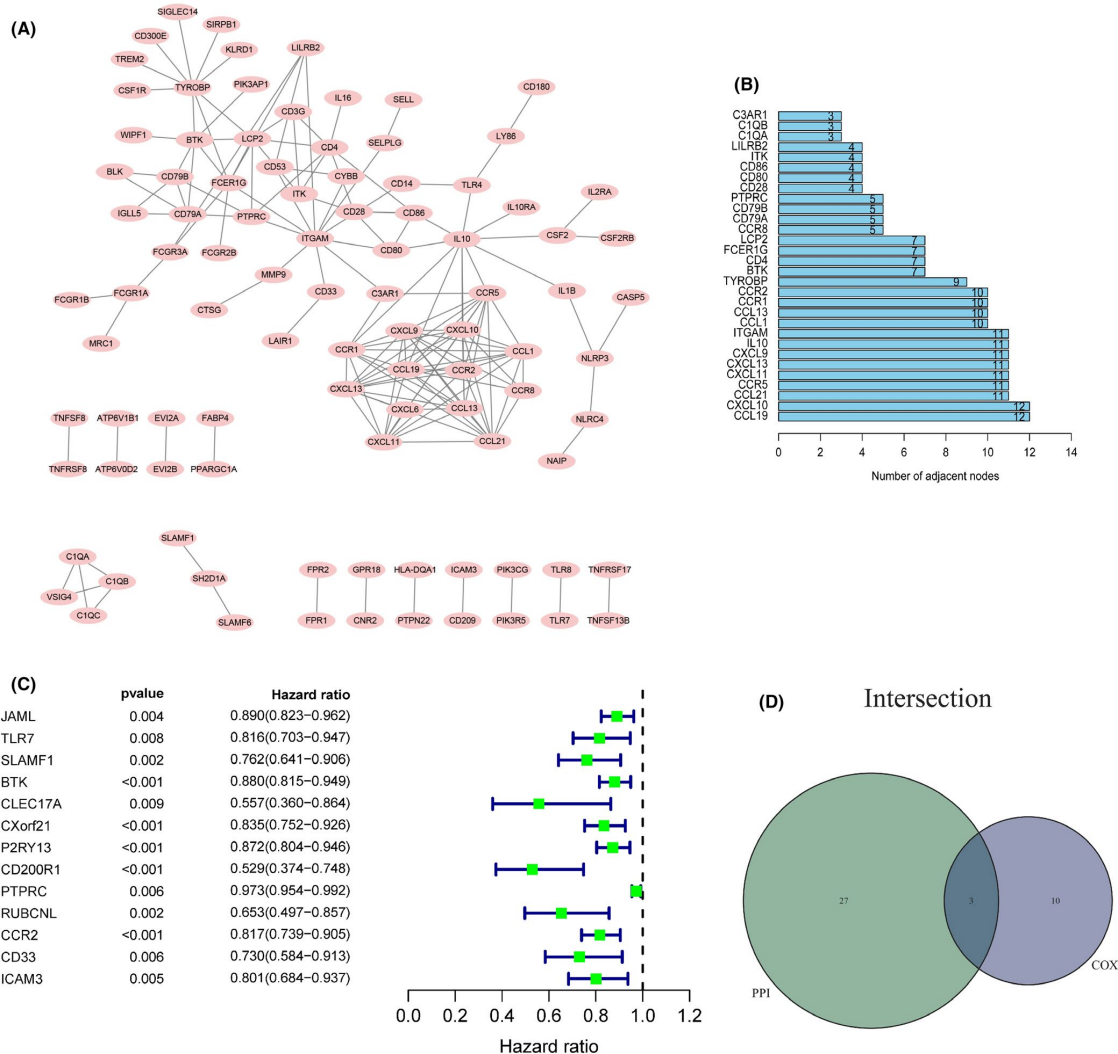
Enrichment plots of PPI and Cox. (A) The minimum interaction requirement was set as high reliability (0.95), and 96 genes constitute an interacting PPI network. (B) The vertical axis means the number of gene nodes, and the horizontal axis indicates the top 30 genes with the maximum number of adjacent nodes in the PPI network. (C) The forest map represented the results of the univariate Cox proportional hazards regression analysis of DEGs. (D) Venn plot showed three common prognostic genes

To study if there were protein interactions among these 374 DEGs, we utilized the Cytoscape software to construct a PPI network based on the STRING database. The 96 genes were selected for a PPI network with a minimum interaction score of 0.95 (Figure 5A). It showed the top 30 genes with the maximum number of adjacent nodes in the bar plot (Figure 5B). The 13 DEGs were shown in the forest map by univariate Cox proportional hazards regression (*p* < 0.01) and Kaplan–Meier analyses (*p* < 0.01) derived from 374 DEGs (Figure 5C and Table S1). Meanwhile, the CCR2, BTK, and PTPRC three DEGs were intersected by the top 30 node count genes in the PPI network and 13 genes in univariate Cox proportional hazards regression analysis (Figure 5D).

### 3.5 CCR2 was selected as the most correlated gene with the survival of LUAD by Lasso regression analysis

To select the functional gene most correlated to LUAD, we carried out Lasso regression analysis on these three genes using the R package "glmnet" and "survival". As shown in Figure S2A, two multigene models were finally generated through the endless selections and simulations of the number of features, the best multigene model (left dotted line), and the simplest multigene model (right dotted line). The gene with the maximum coefficient corresponding to the logarithm λ of the best multigene model was CCR2, which indicated that the expression of CCR2 was the most correlated with the survival of LUAD (Figure S2B).

### 3.6 CCR2 had the potential to be a factor regulating immune‐related activities

**FIGURE 6:**
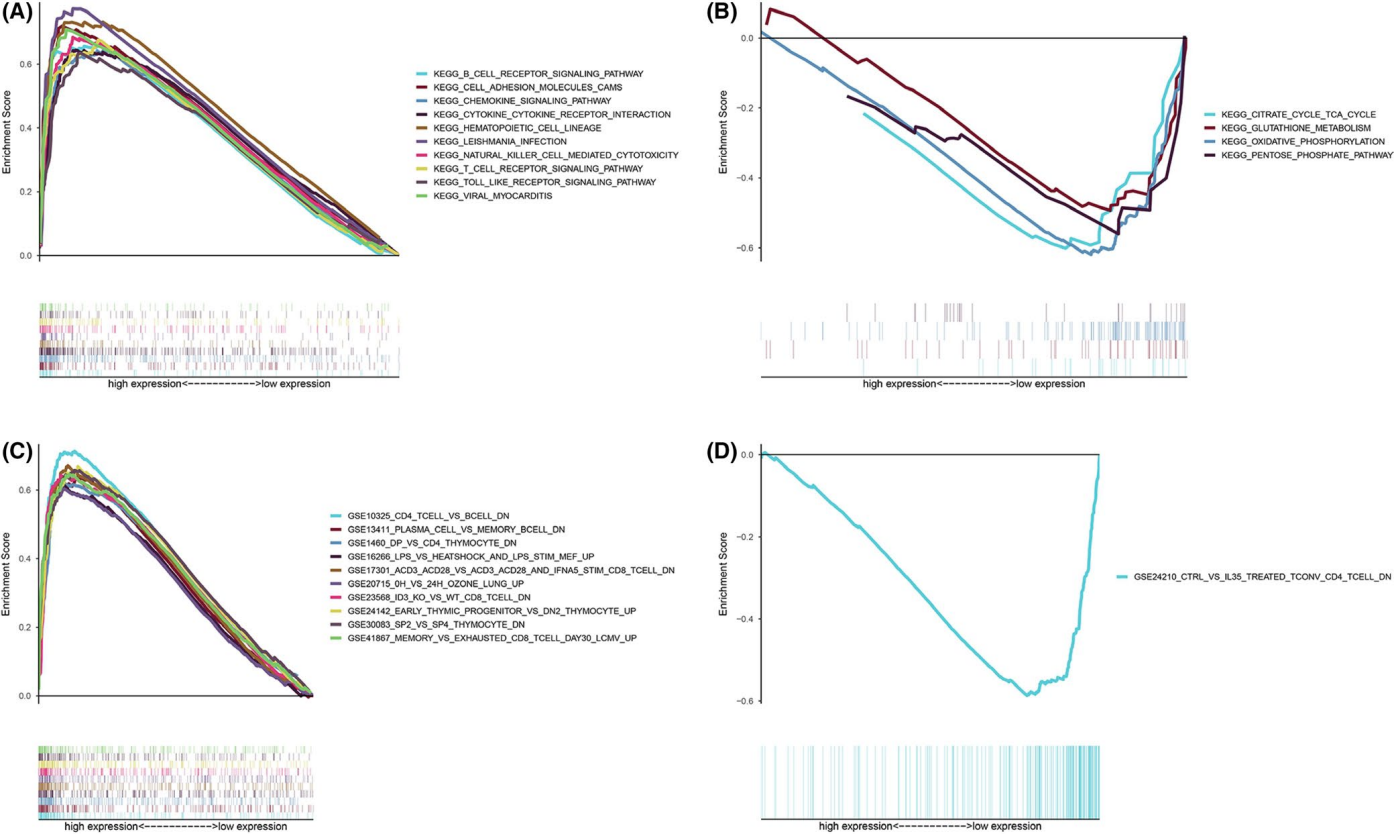
GSEA for samples with high CCR2 expression and low expression. (A) Top 10 enriched gene sets from C2 kegg gene set v7.1 collected by CCR2 high‐expression and (B) low‐expression group. Each pathway was plotted with different color curves. NOM *p* < 0.05 were considered significant. (C) The top 10 enriched gene sets from C7 gene set v7.1 was collected by the CCR2 high‐expression and (D) low‐expression group, and NOM *p* < 0.05 was considered significant

To study the correlation between CCR2 expression with immune‐related activities, CCR2 expression‐associated signal pathways were investigated by GSEA enrichment analysis. Tumor samples were divided into high and low groups by median of CCR2 expression. The results indicated C2 kegg gene sets in CCR2 high‐expression group were primarily enriched in the B cell or T cell receptor signaling pathway, chemokine signaling pathway, and natural killer cell‐mediated cytotoxicity (Figure 6A and Table S2); but enriched in the cell metabolism‐related signaling pathways in CCR2 low‐expression group (Figure 6B and Table S2). Furthermore, multiple C7 immunological gene sets were enriched in the CCR2 high‐expression group (Figure 6C and Table S2). In contrast, only one C7 immunological gene set was enriched in the low‐expression group of CCR2 (Figure 6D and Table S2). These results suggested that CCR2 may be an important factor in regulating immune‐related activities.

### 3.7 The correlation of CCR2 expression with the survival and clinicopathological characteristics

**FIGURE 7:**
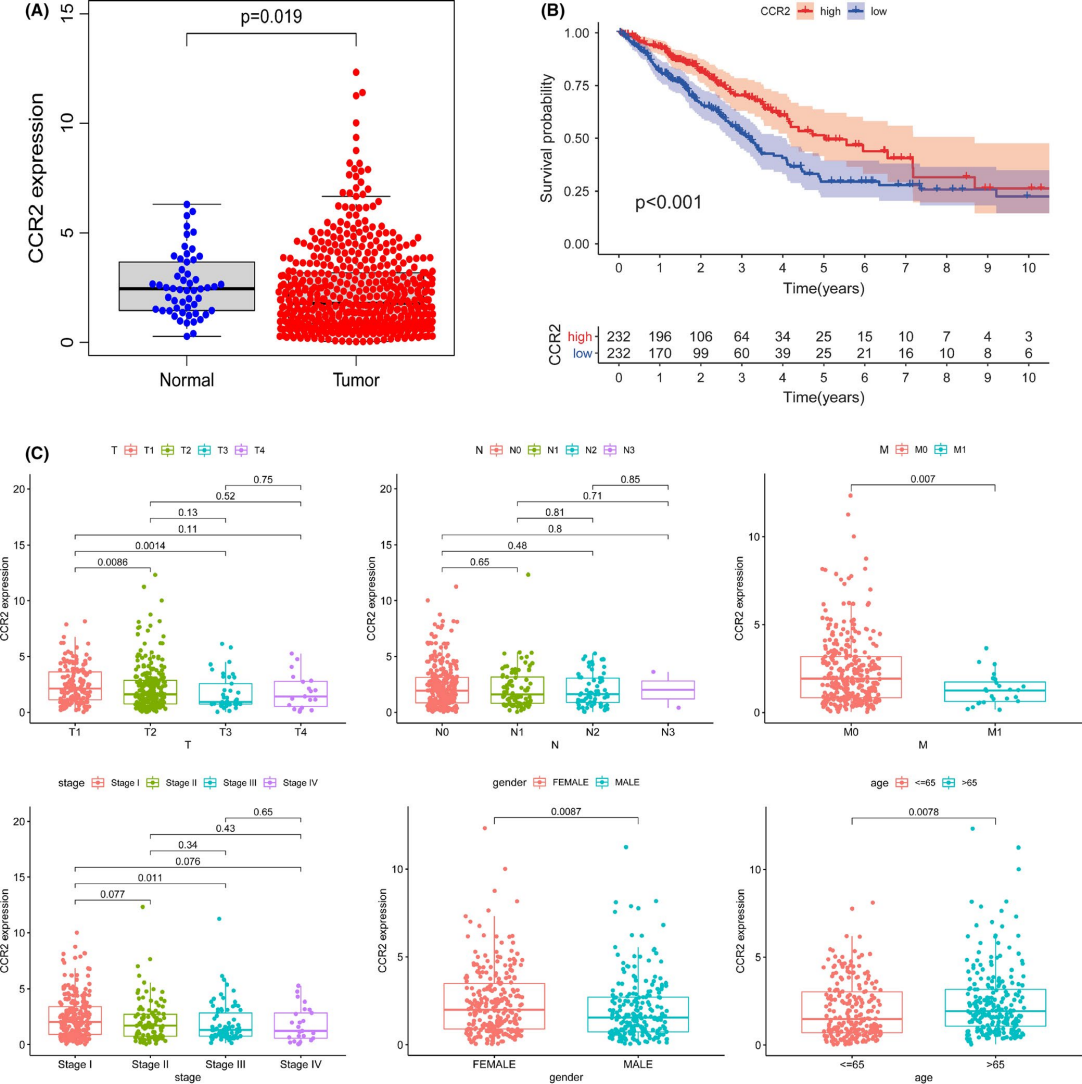
Correlation of CCR2 expression with prognosis in LUAD. (A) Differential expression of CCR2 in normal and tumor cases. All normal and tumor samples were analyzed by the Wilcoxon rank‐sum test and *p* = 0.019. (B) The relationship between CCR2 expression and patient survival rate was analyzed by Kaplan–Meier survival analysis. (C) Wilcoxon rank‐sum and Kruskal–Wallis rank‐sum tests showed that CCR2 expression was significantly correlated with clinicopathological characteristics of LUAD patients

To identify the relationship between CCR2 expression and survival clinicopathological characteristics, we analyzed the expression of CCR2 in LUAD and normal samples, which indicated significantly lower CCR2 expression in LUAD samples than that in normal ones (Figure 7A, *p* = 0.019). All LUAD samples were divided into high and low‐expression groups according to the median of CCR2 expression level. It was indicated that the group with high CCR2 expression had a positive correlation with survival rate (Figure 7B, *p* < 0.001). The expression of CCR2 gradually decreased with the progression of the TNM stages and clinicopathological staging characteristics. Moreover, the expression of CCR2 was correlated to age and gender (Figure 7C).

### 3.8 Correlation between CCR2 expression and TICs

**FIGURE 8:**
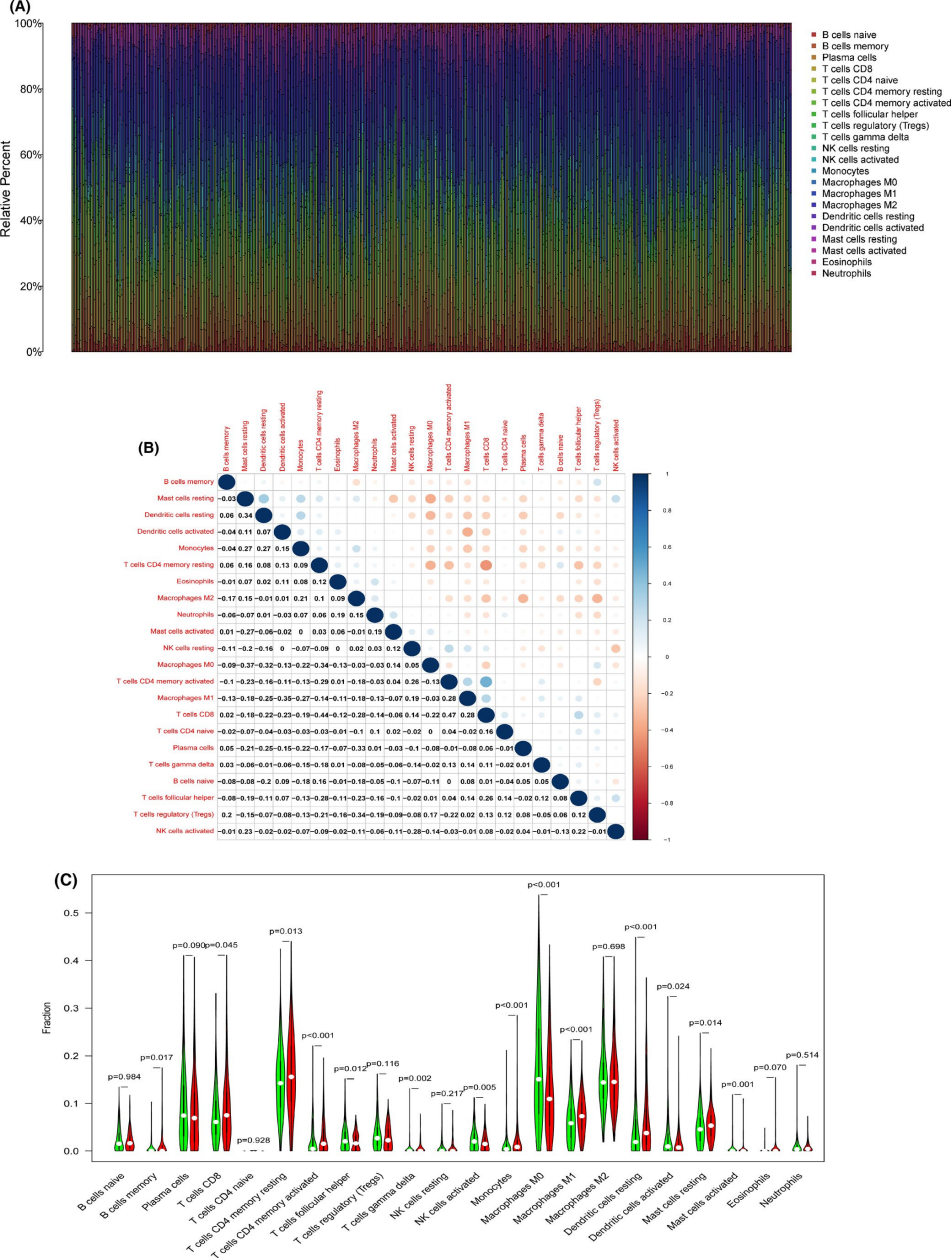
Correlation of CCR2 expression with TICs in LUAD. (A) The bar chart showed a ratio of 21 TICs in each LUAD sample. (B) The heat map showed the relationship between immune cells in the TME. The dots’ size indicated the degree to which they are related, and the color of the dots indicated whether they were positively correlated (blue) or negatively related (red). The number in the figure represented the correlation coefficient between immune cells. Blank squares showed no significance (*p* > 0.05), and the Pearson coefficient was applied for this analysis. (C) All LUAD cases were divided into the high (red) and low (green) CCR2 expression groups, based on the median of CCR2 expression, and the Wilcox rank‐sum test was utilized to analyze the difference between the two groups, *p* < 0.05 was significant

To further identified the correlation between CCR2 expression and TICs, the ratios of immune cells in the TME of each LUAD sample were obtained using the R package "limma" and CIBERSORT algorithm^14^ in R 4.0.2 software. The vertical axis represented the percentages of 22 kinds of immune cells, which visualized the immune cells infiltration results (Figure 8A). It was shown that the correlation between immune cells in Figure 8B. The fraction of 22 kinds of immune cells was shown in the violin diagram with low or high CCR2 expression according to the median of CCR2 expression (Figure 8C and Table S3). The result demonstrated that CD8^+^ T cells, M1 macrophages, and active/resting CD4^+^ T memory cells in the CCR2 high‐expression group were higher than those in the CCR2 low‐expression group. Moreover, the result also proved that CCR2 expression was closely correlated with immune cells in the TME.

## 4 DISCUSSION

TME has a significant correlation with the occurrence and development of lung cancer and has been receiving substantial attention in the immunotherapy of LUAD.^15^, ^16^ The LUAD is a human lung cancer subtype with mutational heterogeneity, which is not only limited to tumor epithelial cells but also spans TME composed of stromal cells and infiltrating immune cells.^17^, ^18^ In this study, the infiltration of immune cells and stromal cells in the TME was calculated, and the results indicated a positive correlation of ImmuneScore with prognosis. We then found the ImmuneScore had a statistically significant difference (*p* < 0.05) through the univariate Cox proportional hazards analysis, but it was a weak factor influencing survival (HR = 0.9998) (Figure S3 and Table S4). Besides, StromalScore and ESTIMATEScore did not affect survival (*p* > 0.05) (Figure S3 and Table S4). In this study, we researched the correlation between immune response and the DEGs, showed that DEGs were correlated with immune‐related activities. Including proliferation, differentiation, activation, and immune response of immune cells in BP; cell membranes and granules in CC; activity and binding of cytokines and receptors in MF through GO analysis. This work was consistent with the results that the cytokine‐cytokine receptor response was highly significant in KEGG enrichment, and reported playing an essential role in developing lung cancer.^19^

Many drugs targeting various components of TME have been approved for clinical therapy, including aromatase, vascular endothelial growth factor (VEGF), and immune checkpoint inhibitors (ICIs), which gained outstanding achievements in the treatment of NSCLC.^20^ However, many NSCLC patients were either resistant to immune checkpoint inhibitors or had immune‐related adverse events.^21^, ^22^, ^23^ For advanced lung cancer, the tumor mutation burden (TMB) can be used as a prognostic biomarker independent of PD‐L1 expression.^24^ Although both PD‐L1 and TMB are widely used as biomarkers for patients’ prognosis, they need more advances to meet clinical immunotherapy. In our present study, CCR2 was finally selected out of DEGs as the most correlated gene with the survival of LUAD by Lasso regression analysis. The low‐expression CCR2 was closely related to the decreased survival rate, clinicopathological features of advanced LUAD. Besides, our univariate Cox proportional hazards analysis results also showed that CCR2 was a vital factor influencing survival (*p* < 0.001 and HR = 0.8185) (Figure S3 and Table S4).

CCR2 is a hybrid receptor.^25^, ^26^ There are two isoforms of CCR2: CCR2A and CCR2B, due to the 50 base pair in the C‐terminal tails.^9^ CCR2B is the dominant kind of CCR2, accounting for 90% of CCR2 expressed.^27^ CCR2 was over‐expressed in breast cancer, pancreatic ductal adenocarcinoma, and prostate cancer, which played a crucial role in tumor metastasis and development by maintaining the hyperplasia and tumor cells’ survival, stimulating the migratory and invasive ability of cancer cells, and inducing inflammatory response and angiogenesis.^28^, ^29^, ^30^ Brummer showed that the CCL2‐CCR2 signaling axis in breast cancer regulated tumor cells’ growth and invasion by regulating tumor angiogenesis, recruiting M2 macrophages, and inhibiting most activation CD8^+^ cytotoxic T cells.^30^ A clinical study of pancreatic ductal adenocarcinoma showed that oral CCR2 inhibitor PF‐04136309 combined with chemotherapy (FOLFIRINOX) could achieve local tumor control in 32 of 33 patients (97%).^31^ Some studies have shown that CCR2 mediates the cellular effect of MCP‐1 to promote the growth and invasion of prostate cancer.^32^ Interestingly, in our research, we found that CCR2 was low‐expression in LUAD, which seemed to elucidate why CCR2 inhibitors have no cytotoxicity on the A549 human lung cancer cell line.^33^ Another study is consistent with our results, showed that infusion of CCR2 gene‐modified effector T cells enhanced anti‐lung cancer response in vivo.^34^

CCR2, with distinct expression modes in LUAD, was different from other cancer types such as breast cancer, pancreatic ductal adenocarcinoma, or prostate cancer.^29^, ^30^ Numerous studies also revealed that CCR2 might play reverse roles in different kinds of tumors, either promoting tumor immune evasion or enhancing the anti‐tumor immune response.^29^, ^30^ In our study, GSEA enrichment analyzed for the correlation of CCR2 expression with immune activity, indicated that some immunological response‐related signaling pathways were active in the CCR2 high‐expression group, such as B cell or T cell receptor signaling pathway, natural killer cell‐mediated cytotoxicity, toll‐like receptor signaling pathway, and chemokine signaling pathway. This result implied that the expression of CCR2 had a positive effect on immune cells’ activating. To further identification of CCR2 in immune function, the correlation with TICs was analyzed. CCR2 has been reported to participate in macrophage polarization regulation in TME, and the absence of CCR2 significantly changed the proportion of M1/M2 macrophages.^35^ Our violin diagram (Figure 8C) showed that CD8^+^ T cells, M1 macrophages, and active/resting CD4^+^ T memory cells in CCR2 high‐expression group were higher than those in the CCR2 low‐expression group, indicated the CCR2 expression was closely correlated with immune cells in the TME. However, M0 macrophages in CCR2 low‐expression group were significantly higher than those in the CCR2 high‐expression group, consistent with the study from Sierra that CCR2 knockout mice exhibited significantly lower polarization of M0 macrophages.^36^ M1 macrophages were responsible for releasing pro‐inflammatory factors to eliminate pathogens during inflammation.^37^ M2 macrophages secrete IL‐10 and TGF‐β, inhibit inflammation, and have many cancer‐promoting functions.^38^ However, our study revealed no significant correlation between CCR2 expression and M2 macrophages (*p* = 0.698), indicated no effect of CCR2 on M2 macrophages in LUAD. Other studies showed a substantial contribution of tumor‐associated macrophages M2 in promoting and transferring tumor cells of NSCLC.^39^ Moreover, macrophages can polarize into M1 (anti‐tumorigenic) or M2 (carcinogenesis) isoforms to play different roles in carcinogenesis.^40^ Therefore, CCR2 might promote M1 rather than M2 macrophages. Taken all together, CCR2 might be an excellent immune indicator for prognosis evaluation in LUAD.

## CONFLICT OF INTEREST

The authors declare that there are no conflicts of interest.

## Supporting information

Figure S1

Figure S2

Figure S3

Table S1

Table S2

Table S3

Table S4

Supplementary Material

Supplementary Material

## Data Availability

All data were obtained from the TCGA database (https://portal.gdc.cancer.gov/). And all final outputs from our analyses are available.
