# Supplementary material for: Prognostic value of CCR2 as an immune indicator in lung adenocarcinoma: A study based on tumor‐infiltrating immune cell analysis
Source: Cancer Med. 2021 May 4;10(12):4150–63. doi: 10.1002/cam4.3931 (PMC8209599; doi:10.1002/cam4.3931)

**SUPPORTING INFORMATION**

**Supplementary Figure**

**Figure S1.** Gene enrichment in different categories and pathways. DEGs in (A) GO and (B) KEGG enrichments were sorted by *q*-value.

**A**

**
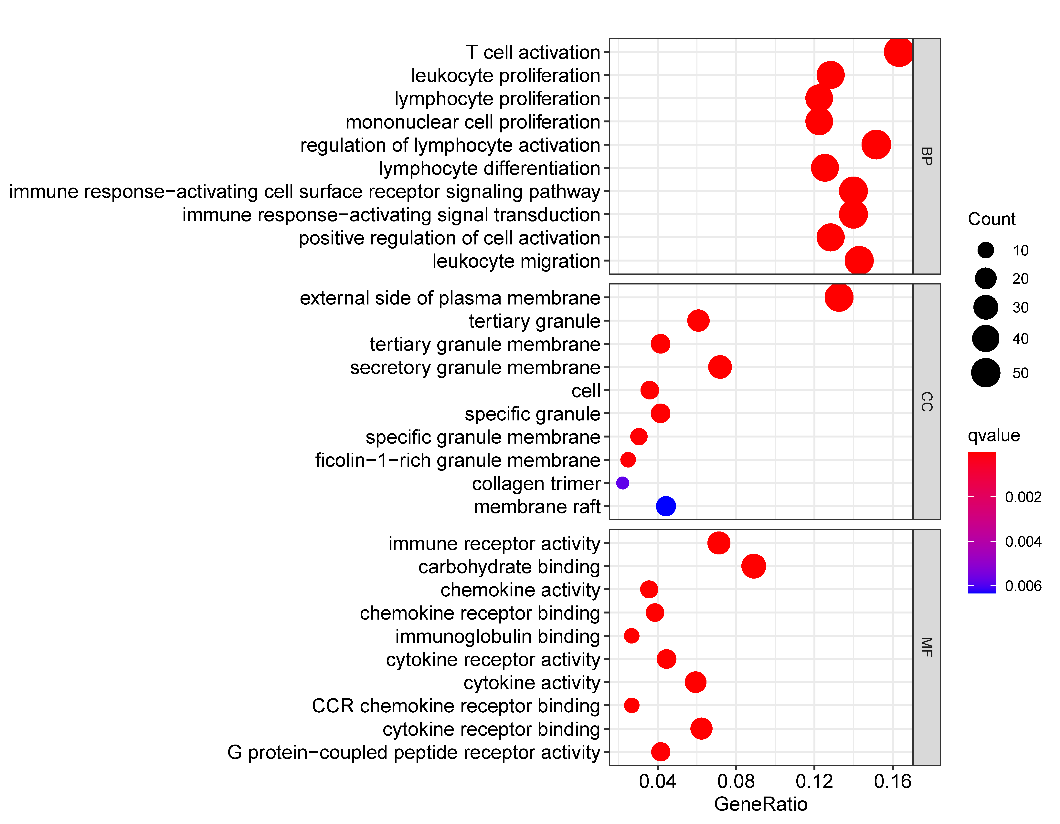
**

**B**


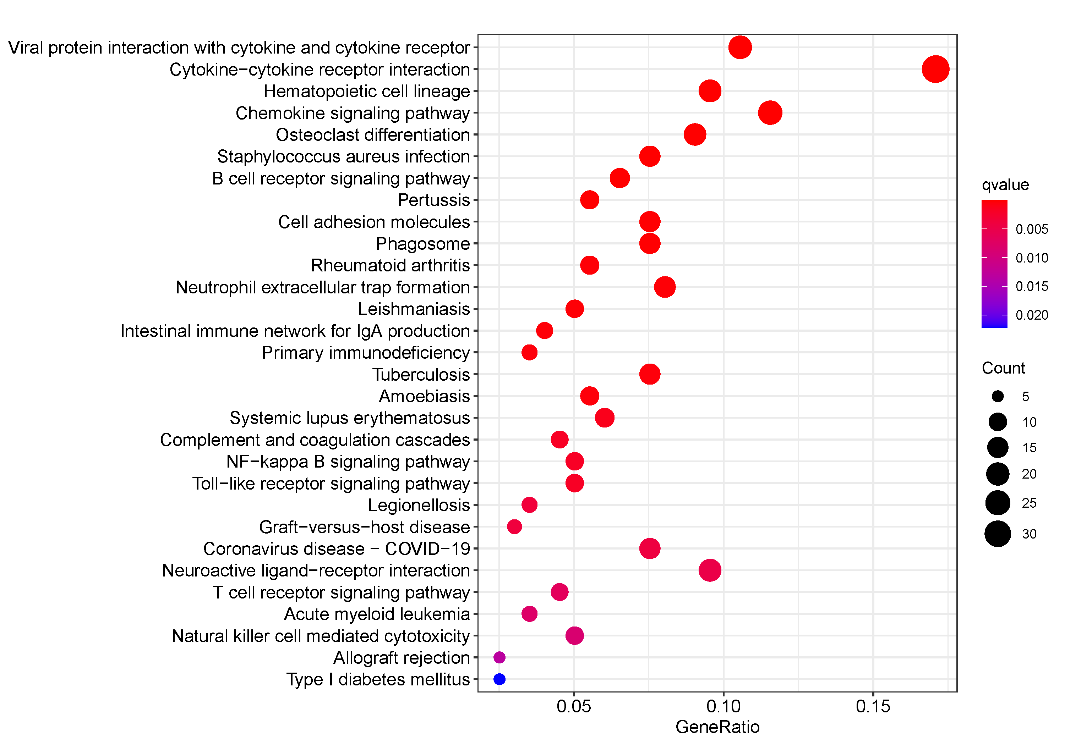

Supplement: Supplementary file 1 — Figure S1 [file CAM4-10-4150-s004.docx]
