# Supplementary material for: Prognostic value of CCR2 as an immune indicator in lung adenocarcinoma: A study based on tumor‐infiltrating immune cell analysis
Source: Cancer Med. 2021 May 4;10(12):4150–63. doi: 10.1002/cam4.3931 (PMC8209599; doi:10.1002/cam4.3931)

**SUPPORTING INFORMATION**

**Supplementary Figure**

**Figure S2.** CCR2 selected by Lasso regression analysis. **(A)** The horizontal axis represented *log ( λ )*, the vertical axis represented cross-validation error, and the upper value represented the number of genes. The graph showed *log ( λ )* and the number of genes with minimum error. **(B)** The chart showed the number and coefficient of selected features in different *λ* states.

**A**

**
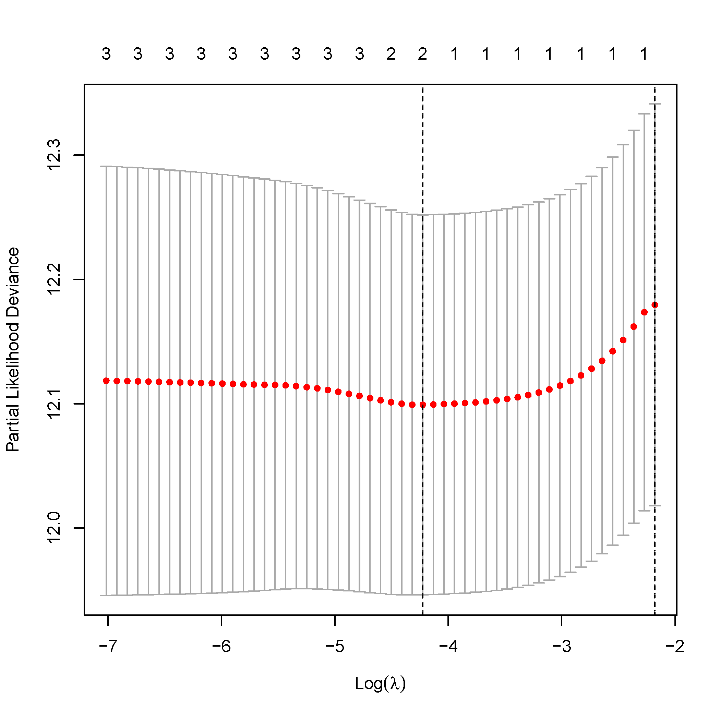
**

**B**


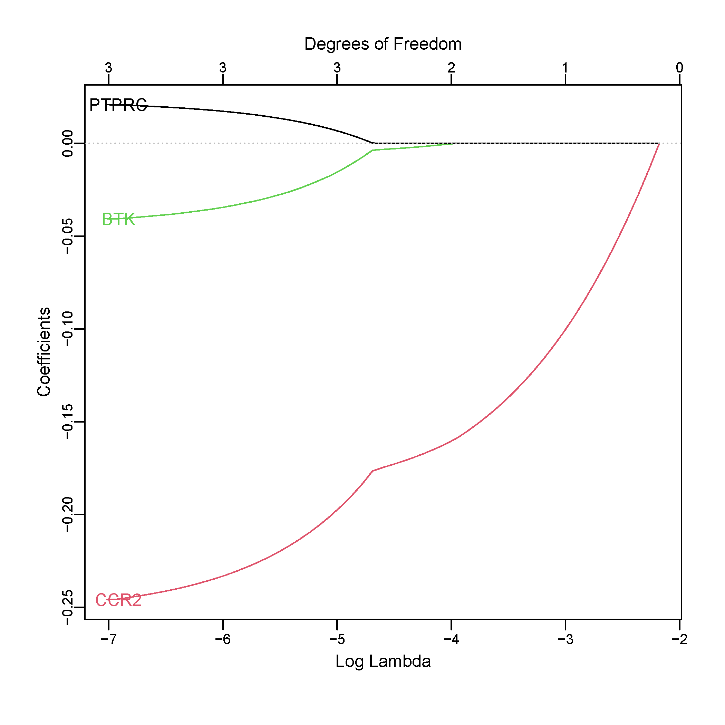

Supplement: Supplementary file 2 — Figure S2 [file CAM4-10-4150-s002.docx]
