# Supplementary material for: Prognostic value of CCR2 as an immune indicator in lung adenocarcinoma: A study based on tumor‐infiltrating immune cell analysis
Source: Cancer Med. 2021 May 4;10(12):4150–63. doi: 10.1002/cam4.3931 (PMC8209599; doi:10.1002/cam4.3931)

**SUPPORTING INFORMATION**

**Supplementary Figure**

**Figure S3.** The association between continuous Immune/Stromal/ESTIMATE Score or CCR2 expression level and survival. The forest map showed the analysis of Immune/Stromal/ESTIMATE Score and CCR2 expression level in LUAD for prognosis prediction using univariate Cox proportional hazards regression.


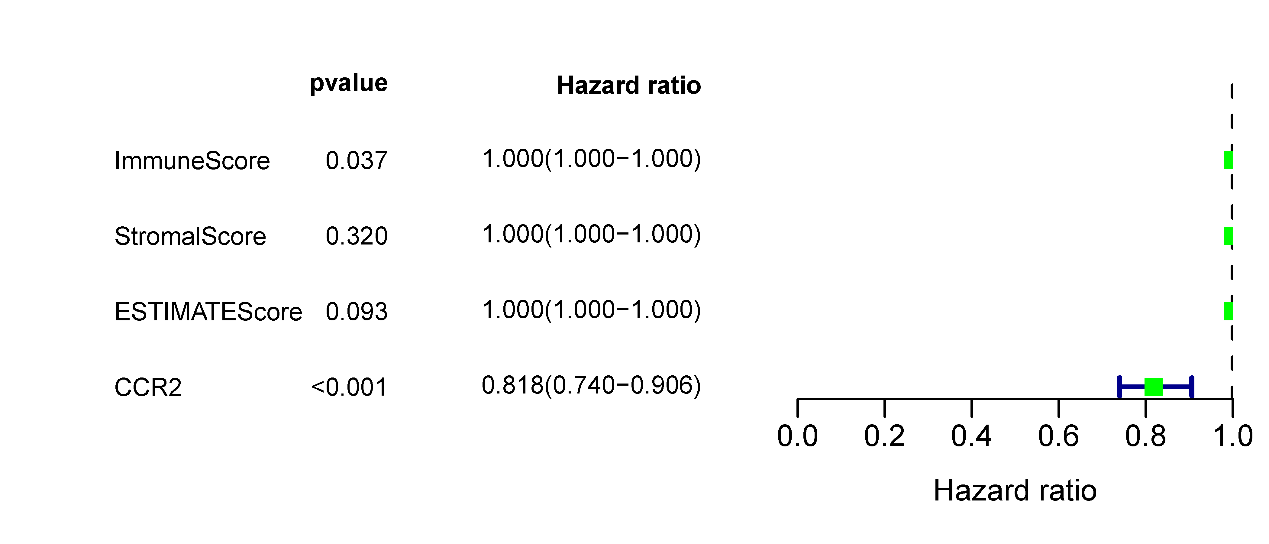

Supplement: Supplementary file 3 — Figure S3 [file CAM4-10-4150-s008.docx]
