# Supplementary material for: Prognostic value of CCR2 as an immune indicator in lung adenocarcinoma: A study based on tumor‐infiltrating immune cell analysis
Source: Cancer Med. 2021 May 4;10(12):4150–63. doi: 10.1002/cam4.3931 (PMC8209599; doi:10.1002/cam4.3931)
Supplement: Supplementary file 4 — Table S1 [file CAM4-10-4150-s007.docx]

**SUPPORTING INFORMATION**

**Table S1. Associations with overall survival and 13 genes in LUAD patients using univariate Cox regression and Kaplan-Meier analyses.**

| gene | HR (95% CI) | *p*-Value | | *q-*Value | |
| --- | --- | --- | --- | --- | --- |
|  |  | Cox | KM | Cox | KM |
| CCR2 | 0.8174（0.7387-0.9045） | 0.0001 | 0.0009 | 0.0179 | 0.0343 |
| CD200R1 | 0.5288（0.3741-0.7476） | 0.0003 | 0.0003 | 0.0387 | 0.0343 |
| CXorf21 | 0.8346（0.7521-0.9261） | 0.0007 | 0.0001 | 0.0517 | 0.0276 |
| BTK | 0.8796（0.8153-0.9489） | 0.0009 | 0.0009 | 0.0517 | 0.0343 |
| P2RY13 | 0.8723（0.8045-0.9458） | 0.0009 | 0.0017 | 0.0517 | 0.0490 |
| RUBCNL | 0.6527（0.4972-0.8570） | 0.0021 | 0.0009 | 0.0599 | 0.0343 |
| SLAMF1 | 0.7619（0.6408-0.9060） | 0.0021 | 0.0042 | 0.0599 | 0.0579 |
| JAML | 0.8899（0.8229-0.9624） | 0.0035 | 0.0020 | 0.0668 | 0.0490 |
| ICAM3 | 0.8007（0.6845-0.9367） | 0.0055 | 0.0075 | 0.0797 | 0.0646 |
| CD33 | 0.7299（0.5837-0.9127） | 0.0058 | 0.0028 | 0.0797 | 0.0512 |
| PTPRC | 0.9729（0.9538-0.9923） | 0.0065 | 0.0083 | 0.0800 | 0.0648 |
| TLR7 | 0.8160（0.7029-0.9474） | 0.0076 | 0.0007 | 0.0800 | 0.0343 |
| CLEC17A | 0.5574（0.3598-0.8636） | 0.0089 | 0.0009 | 0.0800 | 0.0343 |

KM: Kaplan-Meier; HR: hazard ratio; CI: confidence interval.
