# Supplementary material for: Prognostic value of CCR2 as an immune indicator in lung adenocarcinoma: A study based on tumor‐infiltrating immune cell analysis
Source: Cancer Med. 2021 May 4;10(12):4150–63. doi: 10.1002/cam4.3931 (PMC8209599; doi:10.1002/cam4.3931)
Supplement: Supplementary file 5 — Table S2 [file CAM4-10-4150-s009.docx]

**SUPPORTING INFORMATION**

**Table S2. Enriched gene sets.**

| MSigDB collection | Gene set name | NES | NOM *p*-val | FDR *q*-val |
| --- | --- | --- | --- | --- |
| c2.cp.kegg.v7.1.symbols.gmt  CCR2 high expression  CCR2 low expression  c7.all.v7.1.symbols.gmt  CCR2 high expression  CCR2 low expression | KEGG_CELL_ADHESION_MOLECULES_CAMSHALLMARK_COMPLEMENT  KEGG_CHEMOKINE_SIGNALING_PATHWAY  KEGG_NATURAL_KILLER_CELL_MEDIATED_CYTOTOXICITYHALL  KEGG_T_CELL_RECEPTOR_SIGNALING_PATHWAY  KEGG_CYTOKINE_CYTOKINE_RECEPTOR_INTERACTIONHALLMA  KEGG_LEISHMANIA_INFECTION  KEGG_B_CELL_RECEPTOR_SIGNALING_PATHWAY  KEGG_VIRAL_MYOCARDITIS  KEGG_TOLL_LIKE_RECEPTOR_SIGNALING_PATHWAY  KEGG_HEMATOPOIETIC_CELL_LINEAGE  KEGG_PENTOSE_PHOSPHATE_PATHWAY  KEGG_OXIDATIVE_PHOSPHORYLATION  KEGG_CITRATE_CYCLE_TCA_CYCLE  KEGG_GLUTATHIONE_METABOLISM  GSE17301_ACD3_ACD28_VS_ACD3_ACD28_AND_IFNA5_STIM_CD8_TCELL_DN  GSE13411_PLASMA_CELL_VS_MEMORY_BCELL_DN  GSE24142_EARLY_THYMIC_PROGENITOR_VS_DN2_THYMOCYTE_U  GSE1460_DP_VS_CD4_THYMOCYTE_DN  GSE30083_SP2_VS_SP4_THYMOCYTE_DN  GSE10325_CD4_TCELL_VS_BCELL_DGSE13411_PLASMA_CELL_VS_MEMORY_BCELL_DN  GSE41867_MEMORY_VS_EXHAUSTED_CD8_TCELL_DAY30_LCMV_UP  GSE23568_ID3_KO_VS_WT_CD8_TCELL_DN  GSE20715_0H_VS_24H_OZONE_LUNG_UP  GSE16266_LPS_VS_HEATSHOCK_AND_LPS_STIM_MEF_UP  GSE24210_CTRL_VS_IL35_TREATED_TCONV_CD4_TCELL_DN | 2.598  2.537  2.480  2.471  2.466  2.471  2.391  2.354  2.346  2.343  1.884  1.803  1.738  1.719  2.670  2.632  2.670  2.610  2.590  2.583  2.570  2.567  2.566  2.563  2.111 | 0  0  0  0  0  0  0  0  0  0  0.008  0.025  0.028  0.044  0  0  0  0  0  0  0  0  0  0  0 | 0  0  0  0  0  0  0  0  0  0  0.137  0.137  0.138  0.195  0  0  0  0  0  0  0  0  0  0  0.035 |

NES: normalized enrichment score; NOM: nominal *p*-value; FDR: false discovery rate. Gene sets with NOM *p*-value < 0.05 and FDR *q*-value < 0.05 were considered as statistical significance. Only a few enriched gene sets in CCR2 low expression group, gene sets with NOM *p*-value < 0.05 or FDR *q*-value < 0.05 are listed in the table.
