# Supplementary material for: Prognostic value of CCR2 as an immune indicator in lung adenocarcinoma: A study based on tumor‐infiltrating immune cell analysis
Source: Cancer Med. 2021 May 4;10(12):4150–63. doi: 10.1002/cam4.3931 (PMC8209599; doi:10.1002/cam4.3931)
Supplement: Supplementary file 6 — Table S3 [file CAM4-10-4150-s001.docx]

**SUPPORTING INFORMATION**

**Table S3. TICs determined by difference test.**

TICs: tumor-infiltrating immune cells.

| TICs | Difference test (*p*-value) |
| --- | --- |
| B cells memory  T cells CD8  T cells CD4 memory resting  T cells CD4 memory activated  T cells follicular helper  T cells gamma delta  NK cells activated  Monocytes  Macrophage M0  Macrophages M1  Dendritic cells resting  Dendritic cells activated  Mast cells resting  Mast cells activated | 0.017  0.045  0.013  <0.001  0.012  0.002  0.005  0.001  <0.001  <0.001  <0.001  0.024  0.014  0.001 |
