# Supplementary material for: Prognostic value of CCR2 as an immune indicator in lung adenocarcinoma: A study based on tumor‐infiltrating immune cell analysis
Source: Cancer Med. 2021 May 4;10(12):4150–63. doi: 10.1002/cam4.3931 (PMC8209599; doi:10.1002/cam4.3931)
Supplement: Supplementary file 7 — Table S4 [file CAM4-10-4150-s003.docx]

**SUPPORTING INFORMATION**

**Table S4. The univariate Cox regression analysis of Immune/ Stromal/ ESTIMATE Score, and CCR2 expression.**

| variable | HR (95% CI) | *p*-Value | |
| --- | --- | --- | --- |
|  |  | Cox | KM |
| ImmuneScore | 0.9998（0.9996-0.9999） | 0.0373 | 0.0291 |
| StromalScore | 0.9999（0.9999-1.0000） | 0.3204 | 0.0554 |
| ESTIMATEScore | 0.9999（0.9998-1.0000） | 0.0929 | 0.0412 |
| CCR2 | 0.8185（0.7397-0.9057） | 0.0001 | 0.0011 |

KM: Kaplan-Meier; HR: hazard ratio; CI: confidence interval.
